# Editorial: Molecular insights in plant reproductive isolation barriers

**DOI:** 10.3389/fpls.2023.1257823

**Published:** 2023-07-26

**Authors:** Masanobu Mino, Clément Lafon Placette, Takahiro Tezuka

**Affiliations:** ^1^ Graduate School of Agriculture, Osaka Metropolitan University, Sakai, Japan; ^2^ Department of Botany, Charles University, Prague, Czechia

**Keywords:** reproductive isolation barriers, pollen-pistil interaction, hybrid weakness, chromosomal instability, plastid-nuclear incompatibility, hybrid lethality

## Introduction

Clarifying the mechanisms preventing gene flow along the speciation continuum is a fundamental issue for both basic and applied biological sciences. Focusing on the molecular mechanisms underlying reproductive barriers between species may not only allow us to use this knowledge in plant breeding, but identifying the genes behind reproductive isolation may in turn support population genomics studies to understand why reproductive isolation evolved in the first place. A well-known theoretical genetic model exists to explain how postzygotic reproductive isolation can arise, known as the Bateson-Dobzhansky-Muller model (Cutter, 2012), and this model has found empirical support among different taxa, including plants (MacNair and Christie, 1983; Oka, 1988; Bomblies et al., 2007; Chen et al., 2016). These findings have fueled numerous avenues of research into novel genetic architecture and molecular aspects of reproductive isolation barriers. No such unifying genetic model exists to explain how pre-zygotic barriers are formed, likely due to the diversity of mechanisms driving their emergence.

To address and update our knowledge of the molecular aspects of plant reproductive isolation mechanisms, we organized this Research Topic. The research findings and critical reviews presented here have the potential to help address the hybrid problem, a major drawback of plant wide hybridization and introgression breeding (WHIB) programs.

## Postpollination/prezygotic isolation barriers

Pollen-pistil interaction (PPI) contributes to reproductive isolation after pollination. Three checkpoints, the stigma, the style, and the ovule, respectively, reject heterospecific pollen if incongruity and/or incompatibility acts between the egg- and the pollen-parent. While self-incompatibility factors participate in interspecific reproductive barriers in the style, other molecular mechanisms operate in the stigma and ovule. This means that intricate mechanisms for rejecting heterospecific pollen function in PPI. Wang and Filatov summarized recent research on the role of PPI in angiosperm speciation by discussing the molecular systems that operate at each checkpoint and emphasized the importance of extending the research beyond model organisms. This mini review serves as an introduction for readers interested in an overview of the role of PPI in postpollination/prezygotic isolation barriers.

## Postzygotic isolation barriers

Postzygotic isolation barriers consist of hybrid weakness, hybrid necrosis, hybrid lethality (HL), hybrid breakdown, and hybrid sterility. The first three phenomena cover growth defects of the F_1_ hybrid but with varying degrees of phenotypic severity. The growth defects appeared in the F_2_ and subsequent generations are referred to as hybrid breakdown. Hybrid sterility refers to male and/or female gametic defects of the hybrid.

Kim et al. explored a novel rice hybrid weakness phenotype, dark tip embryo (DTE), which is associated with abnormal floral organ development, in an introgression line from an interspecific cross between *Oryza sativa* (japonica) and *O. rufipogon*. A causal gene, *DTE9*, and other unknown genetic factors function coordinately to give rise to DTE. *DTE9* is an allele of *OsMADS8*, a MADS-domain transcription factor gene, and may induce the hybrid weakness phenotype by modifying floral organ identity genes and the signaling and catabolism of abscisic acid. Their study enhances our understanding of the genetic and molecular mechanisms involved in hybrid weakness.

While whole-genome duplication (polyploidization) acts as a major driving force of speciation in plants, the concomitant or independent occurrence of minor chromosome changes, such as aneuploidization, is also important for evolutionary mechanisms (De Storme and Mason, 2014). Lv et al. analyzed a single lineage from synthetic allotetraploid wheat (*S^l^S^l^AA*) derived from an intergeneric cross between *Aegilops longissima* (*S^l^S^l^*) and *Triticum urartu* (AA), which showed a high degree of structural and numerical variation in chromosomes. This lineage derived from a single euploid individual revealed transgenerational chromosomal instability and reduced seed-setting. Thus, genetic/epigenetic mutations of a key gene in the meiosis machinery may be involved in this chromosomal instability. The evidence of perturbed meiosis in the synthetic allotetraploid wheat indicates that factors involved in chromosomal instability affect traits related to reproductive fitness. This study provides useful information for the breeding program of wheat as well as other allopolyploid crops.

Recurrent genetic conflicts between selfish genes, such as transposons and meiotic drive elements, and the host genome contingently provoke hybrid dysfunction (Presgraves, 2010). Genetic factors encoded in cytoplasmically transmitted plastids and mitochondria are considered selfish genes, as they were ancient genetic symbiotes of eukaryotic cells. The role of such selfish genes in reproductive isolation has been found in plastid-nuclear incompatibility (PNI), which reveals asymmetric reproductive isolation in reciprocal crosses of several plant species, including *Silene nutans* (Postel et al., 2022). The hybrids between genetically different lineages of *S. nutans* exhibit strong PNI (seedling lethality), and the candidate gene pairs causing this hybrid defect are a large and a small subunit of the plastid ribosome encoded by plastid and nuPt (nuclear gene targeting plastid protein) genes, respectively. Among many plastid-nuclear gene pairs, Postel et al. analyzed *rps11* (plastid gene) and *rps21* (nuPt gene) because they had the highest number of mutations and subsequently modified the interaction between the two molecules. Based on the relation between variation of amino acid residue centrality, which affects the protein structure network, and variation of PNI, the impact of structural modification of the plastid ribosome on reproductive isolation became apparent in *S. nutans*. The protein crystallographic approach used in this research will help us delineate features that occur in other deleterious protein interactions that cause hybrid problems.

Finally, He et al. reviewed recent advancements in the research of HL and discussed possible methods to overcome it. HL consists of two categories: hybrid seed and seedling lethality. The former is caused by either genetic effects originating from the hybrid embryo or endosperm, while the latter is caused by either autoimmune responses or other molecular systems. In addition, the active use of hybrid lethality was discussed as a means of preventing gene flow from crop cultivars to wild relatives in the context of nature protection. This comprehensive review provides novel insights into the mechanism of HL.

In summary, this Research Topic brings together recent findings and literature to enhance our understanding of the genetic and molecular aspects of plant reproductive isolation barriers. These insights also provide clues to circumvent the hybrid problems in WHIB programs.

## Author contributions

MM: Conceptualization, Writing – original draft. CLP: Writing – review & editing. TT: Writing – review & editing.
